# Hepatitis C Virus, Cryoglobulinemia, and Kidney: Novel Evidence

**DOI:** 10.6064/2012/128382

**Published:** 2012-07-08

**Authors:** Fabrizio Fabrizi

**Affiliations:** Division of Nephrology and Dialysis, Maggiore Hospital and IRCCS Foundation, Pad. Croff, Via Commenda 15, 20122 Milano, Italy

## Abstract

Hepatitis C virus infection can lead to chronic active hepatitis, cirrhosis, and liver failure; however, it is also associated with a wide range of extra-hepatic complications. HCV is associated with a large spectrum of histopathological lesions in both native and transplanted kidneys, and it is increasingly recognized as an instigator of B cell lympho-proliferative disorders including mixed cryoglobulinemia. Mixed cyoglobulinemia is a systemic vasculitis primarily mediated by immune complexes; it is characterized by variable organ involvement including skin lesions, chronic hepatitis, glomerulonephritis, peripheral neuropathy, and arthralgias. The most frequent HCV-associated nephropathy is type I membranoproliferative glomerulonephritis, usually in the context of type II mixed cryoglobulinemia. Various approaches have been tried for the treatment of HCV-related glomerulonephritis, including immunosuppressive therapy (corticosteroids and cytotoxic agents), plasma exchange and antiviral agents. Data on the antiviral treatment of HCV-associated glomerulonephritis are not abundant but encouraging results have been provided. Immunosuppressive therapy is particularly recommended for cryoglobulinemic kidney disease. Recent evidence has been accumulated on rituximab therapy for HCV-related cryoglobulinemic glomerulonephritis exists but several questions related to its use remain unclear. Distinct approaches should be considered for the treatment of HCV-associated cryoglobulinemic glomerulonephritis according to the level of proteinuria and kidney failure.

## 1. Introduction

Cryoglobulinemia is a pathologic condition in which the blood contains immunoglobulins that have the property of reversible precipitation from human serum cooled to 4°C. The discovery in the human serum of proteins which reversibly precipitate in the cold was made in 1933 [1]. In 1947, Lerner and Watson found that these proteins were *γ*-globulins and introduced the term cryoglobulins (cold precipitable serum globulins) [2]. A detailed nosographic placement to the cryoglobulinemic disease within the vast family of systemic vasculitis was made by Meltzer et al. who first described the clinical syndrome of essential mixed cryoglobulinemia (EMC), characterized by purpura, weakness, arthralgias, and, in some patients, organ involvement [3]. On the grounds of immunochemical studies, Brouet et al. identified 3 types of cryoglobulins [4]. In type I, the cryoprecipitable immunoglobulin is a single monoclonal Ig. Types II and III cryoglobulinemias are both mixed types (MC), composed of at least two immunoglobulins. In both of them, a polyclonal immunoglobulin G (IgG) is bound to another Ig which is an antiglobulin and acts as a rheumatoid factor (RF). The main difference between two types of MCs is that in type II, the RF usually of the IgM class is monoclonal, whereas in type III, it is polyclonal. Both components of MCs, IgG, and IgM RF are necessary for precipitation in the cold, whereas the individual components do not have this property. Patients are considered to have a significant cryoglobulin level when it is >0.05 g/L on two determinations. Some laboratories characterize cryoglobulinemia using immunofixation or immunoelectrophoresis, and quantify the cryoglobulin level by determining the cryocrit, as the percentage of the total volume. The use of immunoblotting for immunochemical characterization is a sensitive and specific method allowing a full identification in 98% [5]. When a cryoglobulin is suspected, serum should be kept warm, and tests should be carried out at 37°C. Serum cryoglobulins may also interfere with spurious quantitation of plasma proteins and erythrocyte sedimentation rate, pseudoleucocytosis, pseudothrombocytosis, or pseudomacrocytosis. 

Mixed cryoglobulinemia represent 60% to 75% of all cryoglobulinemias and are found in connective tissue diseases, infectious, or lymphoproliferative disorders, that is, secondary MC. Since the identification of hepatitis C virus [6, 7], many authors have recognized it as the cause of 80% to 90% of MC [8, 9]. HCV is primarily associated with type II MC (which typically has an IgM*k* RF with antiidiotypic activity), and to a lesser extent, with type III MC [10–12]. In the absence of identified etiologic factor (<5% of all MC), cryoglobulinemic vasculitis is defined as essential or idiopathic. This paper aims to describe, main characteristics of HCV-related cryoglobulinemia with a special focus on kidney involvement. 

### 1.1. Epidemiology

Beside chronic liver disease, relevant extrahepatic manifestations of HCV include cryoglobulinemia, lymphoproliferative disorders, and renal diseases (Table 1). Several investigators have given evidence of the association between HCV and glomerular disease in both native [5] and transplanted kidneys [13–15]. A variety of kidney diseases have been associated with HCV. The kidney manifestations of HCV are uncommon, and the available information on their frequency is mostly based on small studies. El-Serag et al. [16] identified 34,204 hospitalized male veterans with HCV (cases) in the US and 136,816 randomly selected patients without HCV (controls) between 1992 and 1999. A significantly greater proportion of HCV-infected patients had porphyria cutanea tarda (0.77% versus 0.06%, *P *< 0.0001), vitiligo (0.17% versus 0.10%, *P *= 0.0002), lichen planus (0.30% versus 0.13%, *P *< 0.0001), and cryoglobulinemia (0.57% versus 0.05%, *P *< 0.0001). There was a greater proportion of membranoproliferative glomeulonerphritis among patients with HCV (0.36% versus 0.05%, *P *< 0.0001), but not membranous glomerulopathy (0.33% versus 0.19%, *P *= 0.86). 

During the last decade, some surveys extracted from large clinical databases have suggested an impact of HCV on incidence (and prevalence) of kidney disease [17–23]. Hepatitis C coinfection was also linked with a significant increase in the risk of HIV-related kidney disease [24, 25]. Patients with HCV might be more likely to have diabetes, obesity, or human immunodeficiency virus, these conditions being independently associated with kidney disease. Chronic HCV is associated with mixed cryoglobulinemia and membranoproliferative glomerulonephritis, and these, in turn, can lead to kidney function impairment [26]. It remains unclear whether and to what extent HCV affects renal function in the whole population. In contrast, four cross-sectional surveys have shown a significant link between HCV and proteinuria in apparent healthy individuals, and the pooled risk of proteinuria in patients with HIV-HCV compared those without HCV coinfection was consistently increased [17, 22, 27, 28].

Multivariate regression models showed that anti-HCV positive rate was significantly associated with proteinuria independently of common metabolic factors, such as diabetes mellitus, arterial hypertension, obesity, and dyslipidemia [28]. Type I MPGN associated with type II MC remains the most common form of kidney disease associated with HCV. Less frequently described kidney lesions are MPGN without cryoglobulinemia and membranous nephropathy. Occasional cases of focal segmental glomerulosclerosis, fibrillary or immunotactoid glomerulopathies, and thrombotic microangiopathy have also been reported [5]. In addition, vasculitis and interstitial nephritis have been associated with HCV. More recent information has been accumulated on the association between HCV and glomerular disease in liver- [29, 30] or kidney/liver [31] transplanted population. The natural history of these HCV-associated nephropathies is characterized by remission and relapsing phases; however, the long-term outcome is not well known.

### 1.2. Pathophysiology of Mixed Cryoglobulinemia Vasculitis

Cryoglobulinemia vasculitis is a systemic vasculitis, that mainly affects the small and, less frequently, medium sized arteries and veins. It is characterized by the deposition of immune complexes containing rheumatoid factor, IgG, HCV RNA, and complement on endothelial surfaces, eliciting vascular inflammation. Mixed cryoglobulinemia is also characterized by the proliferation of B-cell clones producing pathogenic IgM with RF activity. MC represents an example of immune complex vasculitis. Intravascular cryoglobulin precipitation is induced by cold temperature and may involve primarily the skin, the peripheral nerve, and the kidney. A leukocytoclastic reaction is commonly involved in vessel damage of the cutaneous vasculitis. In patients with peripheral neuropathy, nerve pathological analysis often highlights moderate to severe axonal damages (axonal degeneration, differential fascicular loss of axons, signs of demyelinization) associated with a small-size vessel vasculitis (arterioles, venules, and capillaries) and an inflammatory infiltrate composed only of monocytes and T lymphocytes, without necrotizing angiitis [32]. Morphological features observed on kidney biopsies of patients with renal involvement are characterized by an important monocyte infiltrate with double contours of the basement membrane, large eosinophilic and amorphous intraluminal thrombi, evocative of membranoproliferative glomerulonephritis (MPGN). Immunofluorescence study shows intraglomerular subendothelial deposits of IgG, IgM (identical to those of the cryoprecipitates), and complement components. In addition, vasculitis of small renal arteries is present in one-third of patients. Extracapillary crescents are rarely observed. In contrast to cutaneous vasculitis [33], HCV RNA has not been prominently detected in immune complexes in renal lesions and has not been detected in the peripheral neuropathy lesions. 

These findings suggest that various pathophysiological processes are involved in different target organs. The prevalent pathogenetic mechanism of HCV-associated cryoglobulinemic glomerulonephritis is represented by the deposition in the glomerulus of a monoclonal IgM RF with particular affinity for the glomerular matrix, an RF produced by permanent clones of B lymphocytes activated by the virus. Cumulative data suggest that HCV particles may be bound directly or indirectly to B-cells, but HCV rarely infects B-cells [34–36]. Marukian et al. [37] have shown that B-cells lack necessary entry receptors and cannot support replication of HCV. In a minority of cases, immune complexes composed of HCV antigens and anti-HCV IgG antibody might deposit in the glomerular structures, in the absence of a concomitant type II MC with a monoclonal IgM RF, indicating an immune complex glomerulonephritis similar to that observed in patients infected with hepatitis B virus. Such a mechanism could explain the noncryoglobulinemic GN rarely observed by others in patients with chronic HCV [38].

### 1.3. The Role of HCV Itself in Mixed Cryoglobulinemia

Various pieces of evidence support the aetiological role of HCV in mixed cryoglobulinemia. A high proportion of patients with MC and chronic liver disease have serologic evidence of HCV infection [39]. A high prevalence of HCV RNA was detected in the great majority (up to 90%) of patients with type II essential MC [40–42]. An increased concentration (up to 10-fold) of IgG anti-HCV antibody in the cryoprecipitate was measured [41]. The majority of the known HCV antigens (core, E1, E2, NS34, NS4, and NS5) and their corresponding antibodies are described in both cryoprecipitate and vascular lesions in tissue sections [43]. HCV RNA was found in the cryoprecipitate of patients with type II MC concentrated up to 1,000 times the respective levels in supernatants [41, 42].

Hepatitis C virus exerts a chronic stimulus on the immune system, which may lead to the proliferation of B-cell clones producing IgM with RF activity. Charles and Dustin [44] have suggested that specific HCV proteins are necessary for clonal B-cell expansion. High concentrations of HCV envelope protein E2 in vitro stimulate B-cell expansion via interaction with CD81, a known HCV E2 entry factor [45]. IgG-bound HCV specifically drives the clonal expansion of B-cells secreting IgM-RF; upon chronic HCV infection, immune-complexed HCV stimulates the expansion of V_H_-1-69^+^ B-cells, encoding RF WA [44]. These cells become clonally predominant by continued antigenic exposure (usually over a decade or more), independently of T-cell help [44]. The HCV E2-CD81 interactions could result in a lowered B-cell stimulation threshold, facilitating the secretion of various antibodies, including IgM-RF. Clonal B-cells expansions are demonstrable in the intrahepatic lymphocyte infiltrates, in the bone marrow, and in the peripheral blood mononuclear cells [46, 47]. It is still not understood why such an expansion occurs more readily in chronic HCV, compared to other chronic viral diseases such as hepatitis B virus (HBV) or human immunodeficiency virus (HIV). Increased serum B-cell activating factor (BAFF), a TNF-alpha family member required for B-cell survival has been described in HCV-associated MC [48, 49].

In a limited number of patients (<10%), monoclonal B-cell expansion leading to type II MC may evolve into frank B-cell non-Hodgkin-lymphoma. Transformation from polyclonal B-cell proliferation (type III MC) to oligo/monoclonal B-cell proliferation (type II MC) and to the overt malignant lymphoma is a multistep process probably requiring multiple mutagenic events [50, 51]. The duration of B-cell stimulation caused by infectious or other exogenous agents has been also implicated.

### 1.4. Pathogenesis of Kidney Injury in HCV-Associated Mixed Cryoglobulinemia

Some evidence support that the kidney injury due to HCV is mediated by cryoglobulins. Cryoglobulins are deposited in the mesangium during their trafficking in the glomerulus. They can also be seen as intense subendothelial IgM deposits by immunofluorescence. Their nephrotoxicity is related to special affinity of the IgM*k*-RF for cellular fibronectin present in the mesangial matrix [52]. It has been possible to induce, in an experimental mouse model, a membranoproliferative glomerulonephritis (MPGN) similar to human cryoglobulinemic glomerulonephritis by intravenous administration of 37°C solubilized type II cryoglobulins from patients with HCV membrano-proliferative glomerulonephritis [53]. The monoclonal IgM*k*-RF was isolated from such cryoglobulins, separately injected, and able to deposit in the glomerulus; this suggests a special affinity of IgM*k*-RF for the glomerular structures. It needs to be clarified if the deposition of a monoclonal IgM RF in the glomerulus occurs alone or as mixed IgG-IgM cryoglobulin not bound to HCV or as a complex made of HCV, anti-HCV IgG and IgM*k*-RF. Only the RF isolated from cryoprecipitable type II MC had specific affinity; all the other monoclonal RFs are not able to fix fibronectin.

The typical histopathological lesion is a membrano-proliferative (mesangiocapillary) glomerulonephritis [54]. Cryoglobulins can also be deposited in the glomerular capillaries as eosinophilic thrombi, usually associated with vasculitis and fibrinoid necrosis of the glomeruli. Endothelial injury may be an expression of the direct cytopathic activity of the virus. Cryoglobulins may also induce endothelitis via antiendothelial antibody activity and complement activation leading to overexpression of VCAM-1 and subsequent platelet aggregation [54–56]. Immune complexes containing HCV antigens have been observed in the mesangium of patients with cryoglobulinemia leading to mesangial expansion [57]. The presence of HCV-related proteins in the mesangium has been associated with higher proteinuria, possibly reflecting direct mesangial damage of HCV [54]. An increased expression of tolllike receptors has been found in the mesangial cells target of HCV-related MPGN, but not in those of non-HCV MPGN. Mesangial upregulation of toll-like receptors is linked with strong inflammatory activity [58]. 

### 1.5. Clinical Features

Clinically, essential mixed cryoglobulinemia is characterized by the triad of purpura, arthralgias, and weakness. The clinical course of patients with MC is variable: some patients have an indolent course, while others develop vasculitic lesions in various organs. Of particular importance is the development of renal disease, since nephritis represents a hallmark of a severe prognosis. Main extrarenal clinical features of MC include neuropathy, hepatomegaly, sicca syndrome, central nervous system, and gut involvement. Peripheral neuropathy has been mostly described as both a motor and sensory polyneuropathy, mainly distal, and of subacute onset; more rarely, patients may present with the multineuropathy features [59–62]. Less frequently, patients may present with a central nervous system involvement due to cerebral vasculitis [63]. Gastrointestinal manifestations are reported in 7.4% of patients with HCV-MC vasculitis. Abdominal pain, surgical abdomen and/or intestinal bleeding were the main presentation. Patients with gastrointestinal manifestations showed more frequent renal (75% versus 30%; *P *= 0.003) and cardiac involvement (25% versus 2%; *P *= 0.006) and higher cryoglobulin levels (2.2 g/L versus 1.2 g/L; *P *= 0.07) [64]. In many series, the pulmonary involvement was infrequent; however, when pulmonary function was routinely investigated, functional abnormalities related to the immunologic aggression to lung interstitium were seen in 61% of patients [65]. Patients frequently exhibit normal or mild elevation of liver enzymes (60%–70% of cases). Individuals with MC usually show serum positivity for anti-HCV antibodies and HCV RNA in serum. Serum rheumatoid factor (RF), which is positive in 16%–70% of HCV positive patients, is usually increased in the setting of HCV MC; the serum levels of C4 and C1q are usually very low [44]. 

The clinical syndrome of mixed cryoglobulinemia vasculitis can be associated with both type II and III cryoglobulins. In the rheumatologic surveys, patients with type III MC outnumbered those with type II MC [66]; conversely, surveys based on the description of renal involvement revealed a greater prevalence of type II MC, the monoclonal IgM component being mostly IgM*k* [67]. While in the few cases of type III MC with renal involvement the glomerular lesions were variable and nonspecific, in type II MC, in which IgM*k* was the monoclonal component, a specific well-characterized pattern of glomerular disease has been described, called “cryoglobulinemic glomerulonephritis.” 

The amount of circulating cryoglobulins is measured as cryocrit, showing various levels in different patients and in the same patients at various times. The relationship between the severity of the extrarenal and renal manifestations and the cryocrit level is still discussed [68]. The frequency of kidney involvement in MC varies from 8% to 58% of patients. In a minority of cases, the renal disease can be the first and unique presenting manifestation which makes the diagnosis of MC possible. More than half of patients have proteinuria and/or hematuria only [67]. A nephritic syndrome is diagnosed in about 20% of cases. Often both nephrotic and nephritis syndromes are simultaneously present. In 10% of patients, an acute oliguric kidney failure is the first indicator of kidney disease. Arterial hypertension is a frequent symptom, affecting more than 50% of patients at the time of diagnosis. This complication is frequently severe and require intense therapy. In many cases, a malignant hypertension is associated with rapidly progressive nephritis, while in others refractory hypertension is independent of the severity of kidney disease [68]. Signs of MC vasculitis usually precede the renal disease for many years; however, in 29% of cases renal and extrarenal involvement are concurrent [67].

The first clinical manifestation of type II mixed cryoglobulinemia usually appear in the fourth of fifth decade of life [69]. Women outnumber men, and MC incidence varies in different geographical areas [69]. The course of MC is usually characterized by periods of extra renal symptoms alternated with periods of quiescence. The exacerbation of extra renal symptoms is often associated with a flare of the renal disease, but it can occur independently. In many patients, renal disease shows an indolent course, and end-stage renal disease requiring dialysis is rare (<10%); patients with cryoglobulinemic nephritis have a poor prognosis mainly because of a high incidence of infectious, end-stage liver, and cardiovascular diseases [68].

Roccatello et al. [70] included 146 patients with cryoglobulinemic nephritis, of whom 87% (*n *= 127) were HCV positive. Type II cryoglobulins (IgG/IgM*k*) occurred in 74.4% of cases. The remainder had type III cryoglobulins. A diffuse MPGN was the most common histological pattern (83%). Cox regression model showed that age, serum creatinine, and proteinuria at onset of kidney disease were associated independently with a risk for developing severe renal failure at followup. Survival at 10 years was about 30% and cardiovascular disease was the cause of death in more than 60% of patients; additional causes of death included infections (10%), hepatic failure (19%), and neoplasia (3%). Kaplan Meier survival curves were worsened by baseline serum creatinine greater than 1.5 mg/dL. Conflicting results had been found in an older study by Tarantino et al. [68] who enrolled 105 patients and showed that the number of deaths caused by infections (21%) and hepatic failure (19%) approached the number of deaths caused by cardiovascular diseases (29%). These opposite findings have been attributed to different use of antibiotics, antiviral agents, or immunosuppressive drugs. In 151 consecutive HCV RNA-positive patients with MC vasculitis prospectively followed up between 1993 and 2009, baseline factors associated with a poor prognosis were severe liver fibrosis (hazard ratio (HR), 5.31), central nervous system (HR, 2.72), kidney (HR, 1.91), and heart involvement (HR, 4.2). Use of antiviral agents was associated with a good prognosis, whereas treatment with immunosuppressant agents had a negative impact. The 1-year, 3-year, 5-year, and 10-year survival rates (from the MC diagnosis) were 96%, 86%, 75%, and 63%, respectively [71].

### 1.6. Therapy of HCV-Associated Mixed Cryoglobulinemia and HCV-Associated Glomerulonephritis

The discovery of HCV and a better understanding of pathophysiological mechanisms provided the opportunity to control HCV-MC using various approaches: (1) antiviral therapy based on the belief that the underlying infection is driving immune complex formation and resultant vasculitis; (2) B-cell depletion therapy targeting B-cells which produce cryoglobulins, and, (3) non-specific immunosuppressive therapy targeting inflammatory cells present in vasculitic lesions. Potential adverse effects of immunosuppressive therapy with glucocorticoids and cytotoxic drugs on an underlying chronic viral infection are a matter of concern [72–74]. 

#### 1.6.1. Antiviral Therapy

There are no randomized controlled clinical trials (RCTs) regarding the antiviral treatment of HCV-associated glomerulonephritis. HCV-related GN is usually, but not invariably, associated to mixed cryoglobulinemia and only small-sized observational studies exist [24, 38, 70, 75–80] (Table 2). Initial studies were based on monotherapy with conventional or pegylated interferon (IFN) but the combined regimen, pegylated IFN plus ribavirin, has become the gold standard of HCV treatment. The optimal treatment for chronic HCV genotype 1 is now the triple therapy, peg-IFN plus ribavirin and direct-acting antiviral agents (DAA) such as telaprevir or boceprevir [81, 82], according to novel guidelines [83]. However, no evidence has been made on DAA use in patients with kidney impairment [83]. 

Overall, available information shows positive results in terms of remission of proteinuria, hematuria and improvement of serum creatinine as a consequence of HCV RNA clearance from serum, and a decrease in circulating cryoglobulin levels. However, HCV eradication has not been obtained in all patients, and the clinical benefit of antiviral therapy is sometimes transient and restricted to patients with low-grade involvement [72–74]. A meta-analysis of controlled clinical trials compared the efficacy and safety of antiviral versus immunosuppressive therapy (corticosteroids alone or with cyclophosphamide) in patients with HCV-related glomerulonephritis [85]. Proteinuria decreased more after antiviral (monotherapy with standard IFN for at least six months) than immunosuppressive therapy, OR 3.12 (95% CI, 0.72; 13.48; *P *= 0.06) (random-effects model). Unfortunately, both treatment regimens failed to significantly improve renal dysfunction. Of note, in all patients with reduction of proteinuria, an HCV RNA clearance from serum was given at the end of antiviral therapy.

Data on the antiviral treatment in the setting of GN-associated with hepatitis C are limited but encouraging results exist; we found by pooled analysis that the frequency of sustained viral response was 0.42, 95% confidence intervals (95% CI), 0.24; 0.61 (random-effects model), with significant heterogeneity (*P *< 0.0001; *I* ^2^ = 85.5%). As listed in Table 2, the frequency of sustained viral response was higher after combination therapy (pegylated IFN plus ribavirin) than antiviral therapy based on recombinant IFN (alone or with ribavirin). Kidney involvement is frequently associated with negative clinical response; thus, a 48-week course has been recommended. In 72 consecutive patients with symptomatic HCV-associated mixed cryoglobulinemia [80], peg-IFN-*α* plus ribavirin (*n *= 42 patients) permitted to achieve a higher rate of complete clinical (67.5% versus 56.3%) and virologic response (62.5% versus 53.1%) as compared with standard IFN-*α* plus ribavirin (*n *= 32), regardless of HCV genotype and viral load. In multivariate analysis, an early virologic response at month 3 (odds ratio (OR), 3.53) was associated with a complete clinical response of MC vasculitis whereas a glomerular filtration rate (GFR) <70 ml/min (OR 0.18) was negatively linked with a complete clinical response [80]. 

Antiviral therapy of HCV-associated GN shows some shortcomings that must be weighted. Firstly, the impact of antiviral therapy on long-term outcomes of kidney disease is not well known. Secondly, response to antiviral therapy is usually rather slow as it takes several weeks to obtain viral clearance and clinical remission, whereas rapid renal involvement is not uncommon in these patients. Thirdly, interferon-alpha has been reported to exacerbate proteinuria in some patients with underlying glomerulopathies [86]. Finally, use of ribavirin in patients with glomerular filtration rate less than 50 mL per min per 1.73 m^2^ had not been recommended in some guidelines [87] even if preliminary data support ribavirin use in patients with chronic kidney disease and GFR < 50 mL/min/1.73 m^2^ in a cautious and well-monitored setting [76]. 

#### 1.6.2. B-Cell Depletion Therapy

 Given the etiologic role of hepatitis C virus infection in the majority of patients with MC, targeting HCV replication by antiviral treatment (interferon plus ribavirin) should be considered the first-line therapy in HCV-related MC and its complications. Recent evidence has given emphasis to the notion that HCV infection represent the “initiation” step followed by the “perpetuation” step where infections, autoantigens, and cell regulation abnormalities support autoimmunity and B-cell lympho-proliferation. 

 Rituximab (RTX) is a chimeric monoclonal antibody directed to CD20 antigen, a transmembrane protein expressed on pre-B and mature lymphocytes, and is highly effective for *in vivo* B-cell depletion, particularly IgM autoantibody-producing B-cells [72]. Due to its selective activity, rituximab has been used instead of other immunosuppressive approaches such as steroids, cyclophosphamide, plasma exchange, and other cytotoxic drugs. High-dose or prolonged corticosteroid treatment greatly increase morbidity (i.e., myopathy, osteoporosis, major infections) and cyclophosphamide may be hazardous (cytopenia, bladder cancer, and opportunistic infections). 

Rituximab was originally approved for the treatment of low-grade B-cell non-Hodgkin's lymphoma (NHL). More recently, it has been used to treat severe hematologic disorders including pure red cell aplasia, haemolytic anaemia, and posttransplant B-lymphoproliferative disorders. Rituximab is usually give intravenously at 375 mg/m^2^, once a week for one month. Mixed cryoglobulinemia is featured by chronic stimulation of B lymphocytes by HCV and widespread autoantibody production related to HCV-induced lowering of the cell activation threshold. Thus, MC patients have been considered appropriate candidates for rituximab therapy. Various reports have emphasized its efficacy in the management of symptomatic mixed cryoglobulinemia [88–106]. RTX proved to have great efficacy for the main vasculitic signs (remission of purpura, arthralgias, and improvement of peripheral neuropathy), even if a relapse of mixed cryoglobulinemia has been noted in many patients after completion of therapy. Laboratory features, that is, reduction of cryocrit percentage and RF activity, and increases of C4 levels were consistent with the clinical efficacy. 

Two uncontrolled pilot trials have been conducted on RTX use for HCV-associated GN. A total of 11 individuals have been treated. An important decrease in proteinuria was noted by Roccatello et al. (2.18 ± 1.57 versus 0.63 ± 0.4 g/day, *P *= 0.04) [92] and Quartuccio et al. (1.74 ± 1.26 versus 0.5 ± 0.729 g/day, *P *= 0.09) [93]. A concomitant reduction of serum levels of rheumatoid factor was also found in the first (751.1 ± 66 versus 363.8 + 273 IU/L, *P *= NS) and second study (1,888.8 ± 1,80 IU/L versus 1,414 ± 2.52 IU/L, *P *= NS). No acute or delayed severe adverse effects were seen. However, clinical relapses of glomerular disease after completion of rituximab therapy were found. 

A number of side effects have been reported in patients with HCV-associated mixed cryoglobulinemia treated with rituximab. RTX has no efficacy on HCV viral clearance and an increment of HCV RNA in both unfractionated sera and the cryoprecipitate has been reported by various authors [88]. Reactivation of liver disease related to HBV- or HCV-infections after rituximab therapy has been also shown. We recently reported on a renal transplant recipient with chronic and mild hepatitis C who received standard rituximab therapy for gastric lymphoma [107]. Rituximab was complicated by cholestatic hepatitis C with extremely high HCV RNA levels; liver failure occurred. The patient developed bacterial pneumonia, and respiratory insufficiency was the cause of death. Although other mechanisms could not be excluded, we implicated rituximab in the pathogenesis of cholestatic hepatitis C in our patient. Randomized controlled trials with adequate size and followup are warranted to clarify the risk to benefit ratio for RTX use in HCV-associated mixed cryoglobulinemia patients, particularly those with kidney involvement. 

In order to better define the place of rituximab in the therapeutic strategy of HCV-related mixed cryoglobulinemia, some authors have used antiviral therapy with or without rituximab in HCV MC. Two prospective, controlled clinical trials have compared the efficacy and safety of a combination of rituximab followed by peg-IFN-*α*/ribavirin versus peg-IFN-*α*/ribavirin for HCV-related mixed cryoglobulinemia. Compared with peg-IFN-*α*/rituximab (*n *= 55), Saadoun et al. [84] observed that rituximab plus peg-IFN-*α*/ribavirin (*n *= 38) had a shorter time to clinical remission (5.4 ± 4 versus 8.4 ± 4.7 months, *P *= 0.004) and higher rates of cryoglobulin clearance (68.4% versus 43.6%, *P *= 0.001). They observed a significant reduction of daily proteinuria and hematuria in both the groups; patients treated with rituximab followed by peg-IFN-*α*/ribavirin also had a significant improvement of kidney function (217.5 ± 47.4 versus 136.9 ± 27 *μ*mol/L, *P *= 0.03), whereas no significant change was noted in the second group (150 ± 30 versus 169.2 ± 44 *μ*mol/L, NS). In the work by Dammacco et al. [108], a complete response was achieved in 54.5% (12/22) of patients who received RTX followed by peg-IFN-*α*/ribavirin and in 33.3% (5/15) of patients on peg-IFN-*α*/ribavirin. No significant change in serum creatinine and daily proteinuria in both the groups occurred. 

#### 1.6.3. Nonspecific Immunosuppressive Agents

Immunosuppressive agents have been given to MC patients with severe disease manifestations such as membranoproliferative glomerulonephritis, severe neuropathy and other life-threatening complications. A combination of corticosteroids and immunosuppressant such as cyclophosphamide and azathioprine has been used while awaiting the generally slow response to antiviral treatments. In a large retrospective study of 105 patients with cryoglobulinemia vasculitis associated renal disease, 80% were administered corticosteroids and/or cytotoxic agents, while 67% underwent plasma exchange [68]. Despite this aggressive approach, long-lasting remission of the renal disease was achieved in only 14% of cases, and the 10-year survival rate was only 49%.

Corticosteroids, used alone or in addition to IFN-*α* did not favourably affect the response of HCV-related vasculitis manifestations in two controlled studies [109, 110]. In one randomized trial, methyl-prednisolone alone given for one year was associated with clinical response in 22% of patients, compared with 66% and 71% in patients receiving IFN-*α* or IFN-*α* plus methyl-prednisolone, respectively [110]. Low dose corticosteroids may help to control minor intermittent inflammatory signs such as arthralgia but do not succeed in cases of major organ involvement (i.e. neurologic, renal) or in the long-term control of MC vasculitis.

Plasma exchange offers the theoretical advantage of removing the pathogenic cryoglobulins from the circulation in order to avoid the rebound increase in cryoglobulinemia commonly seen after discontinuation of corticosteroids. When used in combination with HCV treatment, three times weekly plasma exchange did not modify the viral response if standard IFN-*α* was given after each plasma exchange session [111]. 

#### 1.6.4. Treatment of HCV-Associated Mixed Cryoglobulinemia: Conclusions

Given to the lack of randomized controlled trials, it is difficult to define a standard strategy for the therapy of HCV-related mixed cryoglobulinemia. We recommend an approach tailored on the disease manifestations, and comorbidities. Antiviral therapy with Peg-IFN plus ribavirin is suggested for HCV-MC patients with mild to moderate disease severity and activity (i.e., without rapidly progressive nephritis, motor neuropathy, or other life-threatening complications). In patients presenting with severe, rapidly progressive vasculitic complications (i.e., progressive renal disease, progressive motor neuropathy, extensive skin disease including ulcers and distal necrosis), a combined treatment with high-dose steroids, immunosuppressors and plasma-exchange has been recommended. In the setting of severe/active but not life-threatening complications RTX may now represent the first option. Novel approaches include antiviral regimens with better efficacy and tolerance, and the combination of rituximab plus antiviral agents, provided no new safety concerns are raised using this approach. 

## Figures and Tables

**Table 1:** Extrahepatic manifestations of HCV infection.

Source	
Kidney	Membranoproliferative GN, membranous nephropathy, focal segmental sclerosis, fibrillary GN, immunotactoid nephropathy, IgA nephropathy, tubulointerstitial nephritis, thrombotic microangiopathy
Skin	Purpura, ulcers; lichen planus; porphyria cutanea tarda
Lung	Alveolitis; pulmonary fibrosis
Joints	Arthralgias; nonerosive arthritis
Thyroid	Autoimmune thyroiditis; hypothyroidism; thyroid cancer
Bone marrow	Monoclonal gammopathies; B-cell lymphoma
Nerves	Peripheral neuropathy; mononeuritis
Muscles	Polymyositis
Glands	Xerostomia; xeroftalmia

**Table 2:** Antiviral treatment of HCV-associated GN: clinical studies.

Authors	SVR	Antiviral therapy, schedule	Reference year
Mazzaro et al. [75]	14% (1/7)	Lymphoblastoid-IFN	2000
Bruchfeld et al. [76]	71% (5/7)	IFN-*α*-2b + ribavirin (*n* = 5)	2003
		Peg-FN-*α*-2b + ribavirin (*n* = 2)	
Rossi et al. [79]	100% (3/3)	IFN-*α*-2b + ribavirin	2003
Alric et al. [26]	67% (12/18)	IFN-*α* + ribavirin (*n* = 14)	2004
		Peg-FN-*α* + ribavirin (*n* = 4)	
Saadoun et al. [80]	59% (13/22)	IFN-*α*-2b + ribavirin (*n* = 12)	2006
		Peg-FN-*α*-2b + ribavirin (*n* = 10)	
Roccatello et al. [70]	11% (6/55)	IFN (*n* = 10)	2007
		IFN + ribavirin (*n* = 45)	
Garini et al. [77]	75% (3/4)	IFN-*α* + ribavirin (*n* = 2)	2007
		Peg-FN-*α*-2a + ribavirin (*n* = 2)	
Abbas et al. [78]	13% (4/30)	IFN-*α* + ribavirin	2008
Saadoun et al. [84]	40% (4/10)	Peg-IFN-*α*-2b + ribavirin	2010
Fabrizi et al.	0.42 (95% CI, 0.24; 0.61)	Pooled analysis	2012

Results have been calculated according to an intention-to-treat (ITT) analysis.

SVR: sustained virological response.

## Abbreviations

CI: Confidence intervals
DAA: Direct-acting antiviral agents
EMC: Essential mixed cryoglobulinemia
GFR: Glomerular filtration rate
HR: Hazard ratio
HBV: Hepatitis B virus
HCV: Hepatitis C virus
HIV: Human immunodeficiency virus
Ig: Immunoglobulin
MC: Mixed cryoglobulinemia
MPGN: Membranoproliferative glomerulonephritis
NHL: Non-Hodgkin's lymphoma
NS: Not significant
OR: Odds ratio
RF: Rheumatoid factor
RCT: Randomized controlled trial
RTX: Rituximab
SVR: Sustained virological response.
